# Mental Health Effects of COVID-19 in Older Adults Are Moderated by Existing Mental Health Needs and Emotional Support

**DOI:** 10.1093/geroni/igab046.357

**Published:** 2021-12-17

**Authors:** Tianyin Liu, Shiyu Lu, Terry Y S Lum, Walker Siu Hong Au, Man-Man Peng, Dara K Y Leung, Gloria H Y Wong

**Affiliations:** The University of Hong Kong, Hong Kong, Hong Kong

## Abstract

Mitigating mental health consequences is one of the priorities for the society to advance, and the aim of this study is to investigate the mental health effects of COVID-19 in older adults and to explore risk and protective factors. Social workers recruited 722 older adults living in the community (mean age 73.6±8.4) from January 2020 to February 2021 in Hong Kong, and interviewed them for basic demographics (age, gender, education, and living status), mental health service use in recent three months (proxy for existing needs), number of people to talk to when feeling down (proxy for emotional support network), and assessed their depression using Patient Health Questionnaire-9 (PHQ-9). Impacts of COVID-19 were indicated by local daily effective reproductive number (Rt) and Nth wave since the start of the pandemic. Generalized linear models (GLM) were applied. Basic demographics were not associated with depression, existing mental health needs (β=2.99, p<.001), Rt (β=1.08, p<.05) and Nth wave (β=0.49, p<.05) were positively associated with depression, while emotional support network was negatively associated with depression (β=-2.47, p<.001). There was also a significant interaction between Rt and Nth wave on depression (β=0.69, p<.05), suggesting ongoing COVID-19 took a toll on older adults’ mental health. Three-way interactions between COVID-19 Rt, Nth wave and existing mental health needs (β=0.25, p<.05) and emotional support network (β=-0.12, p=.07) on depression further indicated that older adults with existing mental health needs warrant more attention, and wider emotional support network may buffer the impact of the pandemic on mental health.

